# Functional Characterization and Direct Comparison of Influenza A, B, C, and D NS1 Proteins *in vitro* and *in vivo*

**DOI:** 10.3389/fmicb.2019.02862

**Published:** 2019-12-17

**Authors:** Aitor Nogales, Teresa Aydillo, Gines Ávila-Pérez, Alba Escalera, Kevin Chiem, Richard Cadagan, Marta L. DeDiego, Feng Li, Adolfo García-Sastre, Luis Martínez-Sobrido

**Affiliations:** ^1^Department of Microbiology and Immunology, School of Medicine and Dentistry, University of Rochester, Rochester, NY, United States; ^2^Centro de Investigación en Sanidad Animal, Madrid, Spain; ^3^Department of Microbiology, Icahn School of Medicine at Mount Sinai, New York, NY, United States; ^4^Global Health and Emerging Pathogens Institute, Icahn School of Medicine at Mount Sinai, New York, NY, United States; ^5^Department of Molecular and Cell Biology, Centro Nacional de Biotecnología, Consejo Superior de Investigaciones Científicas, Madrid, Spain; ^6^Department of Veterinary and Biomedical Sciences, South Dakota State University, Brookings, SD, United States; ^7^Department of Medicine, Division of Infectious Diseases, Icahn School of Medicine at Mount Sinai, New York, NY, United States; ^8^The Tisch Cancer Institute, Icahn School of Medicine at Mount Sinai, New York, NY, United States

**Keywords:** influenza A virus (IAV), influenza B virus (IBV), influenza C virus (ICV), influenza D virus (IDV), non-structural protein 1 (NS1), signal transducer and activator of transcription 2 (STAT2), innate immunity, interferon (IFN)

## Abstract

Influenza viruses are important pathogens that affect multiple animal species, including humans. There are four types of influenza viruses: A, B, C, and D (IAV, IBV, ICV, and IDV, respectively). IAV and IBV are currently circulating in humans and are responsible of seasonal epidemics (IAV and IBV) and occasional pandemics (IAV). ICV is known to cause mild infections in humans and pigs, while the recently identified IDV primarily affect cattle and pigs. Influenza non-structural protein 1 (NS1) is a multifunctional protein encoded by the NS segment in all influenza types. The main function of NS1 is to counteract the host antiviral defense, including the production of interferon (IFN) and IFN-stimulated genes (ISGs), and therefore is considered an important viral pathogenic factor. Despite of homologous functions, the NS1 protein from the diverse influenza types share little amino acid sequence identity, suggesting possible differences in their mechanism(s) of action, interaction(s) with host factors, and contribution to viral replication and/or pathogenesis. In addition, although the NS1 protein of IAV, IBV and, to some extent ICV, have been previously studied, it is unclear if IDV NS1 has similar properties. Using an approach that allow us to express NS1 independently of the nuclear export protein from the viral NS segment, we have generated recombinant IAV expressing IAV, IBV, ICV, and IDV NS1 proteins. Although recombinant viruses expressing heterotypic (IBV, ICV, and IDV) NS1 proteins were able to replicate similarly in canine MDCK cells, their viral fitness was impaired in human A549 cells and they were highly attenuated *in vivo*. Our data suggest that despite the similarities to effectively counteract innate immune responses *in vitro*, the NS1 proteins of IBV, ICV, or IDV do not fully complement the functions of IAV NS1, resulting in deficient viral replication and pathogenesis *in vivo*.

## Introduction

Influenza viruses belong to the *Orthomyxoviridae* family and are enveloped viruses which contain a segmented genome of single-stranded RNA molecules of negative polarity (Wright et al., 2007; Nogales and Martinez-Sobrido, 2016; Martinez-Sobrido et al., 2018; Blanco-Lobo et al., 2019). Currently, there are four recognized influenza virus types: A, B, C, and D (IAV, IBV, ICV, and IDV, respectively) (Wright et al., 2007; Chen and Holmes, 2008; Wanitchang et al., 2012; Tong et al., 2013; Baker et al., 2014; Yoon et al., 2014; Hengrung et al., 2015; Matsuzaki et al., 2016; Wang et al., 2016; Foni et al., 2017; Nogales et al., 2017c; Su et al., 2017; Nakatsu et al., 2018; Asha and Kumar, 2019; Zhang et al., 2019). IAV and IBV contain eight genomic viral (v)RNA segments (Wright et al., 2007), and two major glycoproteins in the virion surface, the hemagglutinin (HA) and neuraminidase (NA), which are responsible for viral binding and release, respectively, of the virus from infected cells (Wright et al., 2007). Moreover, HA and NA glycoproteins are also the major antigenic determinants of IAV and IBV and they are used to further classify them in subtypes (IAV) or lineages (IBV) (Martinez-Sobrido et al., 2018; Blanco-Lobo et al., 2019). IAV have a broad species tropism, infecting multiple avian and mammalian species, including humans (Parrish et al., 2015; Mostafa et al., 2018; Long et al., 2019), while IBV are primarily limited to infect humans (Osterhaus et al., 2000; Chen and Holmes, 2008; Piepenbrink et al., 2019). IAV and IBV are both responsible of seasonal epidemics in the human population and are considered a major public health and economic concern worldwide (Krammer et al., 2015; Raviotta et al., 2017; Federici et al., 2018; Paules et al., 2018). In contrast, the genome of ICV and IDV is made of seven vRNA segments, since the functions of the HA and the NA glycoproteins in IAV and IBV are combined in the hemagglutinin-esterase-fusion (HEF) glycoprotein of ICV and IDV (Hengrung et al., 2015; Matsuzaki et al., 2016; Wang et al., 2016; Nakatsu et al., 2018; Asha and Kumar, 2019; Zhang et al., 2019). ICV causes mild respiratory illness in humans and pigs and is not thought to cause epidemics (Matsuzaki et al., 2016). On the other hand, IDV principally affects cattle and pigs and, to date, IDV is not known to infect humans (Foni et al., 2017; Su et al., 2017; Asha and Kumar, 2019). Concerns associated with influenza virus are further exacerbated by their ability to efficiently transmit by the respiratory route and the limited antiviral therapeutic options for their treatment (Munster et al., 2009; Steel et al., 2009a; Seibert et al., 2010; Kimble et al., 2011; Herfst et al., 2012; Fouchier et al., 2013; Watanabe and Kawaoka, 2015; Cheng and Subbarao, 2018; Federici et al., 2018; Nogales et al., 2018c; Paules et al., 2018).

Host innate immune responses activated upon infection, limit viral replication and dissemination (Randall and Goodbourn, 2008; Carrero, 2013; Nogales et al., 2018b). Consequently, viruses have developed multiple mechanisms to counteract the host antiviral responses, especially the induction of interferon (IFN) and the activities of IFN-stimulated gene (ISG) proteins that restrict virus replication (Wang et al., 2007; Randall and Goodbourn, 2008; Iwasaki and Pillai, 2014; Nogales et al., 2018b). Influenza virus non-structural protein 1 (NS1) is a multifunctional protein, but one of its major and conserved function relates to its ability to inhibit host innate immunity, allowing efficient viral replication in IFN-competent systems (Garcia-Sastre et al., 1998; Talon et al., 2000; Dauber et al., 2006; Kochs et al., 2007; Hai et al., 2008; Hale et al., 2008a, b; Richt and Garcia-Sastre, 2009; Steel et al., 2009b; Steidle et al., 2010; Pachler and Vlasak, 2011; Pica et al., 2012; Dankar et al., 2013; Ayllon et al., 2014; DeDiego et al., 2016; Nogales et al., 2016b, 2017b, 2018a,b; Chauche et al., 2018; Rodriguez et al., 2018). NS1 is the primary transcript from the viral non-structural (NS) genome segment 8 (IAV and IBV) or 7 (ICV and IDV), which also encode the nuclear export protein (NEP) by using an alternative splicing mechanism (Lamb et al., 1980; Nakada et al., 1986; Muraki et al., 2010; Robb et al., 2010; Chua et al., 2013; Dela-Moss et al., 2014).

Studies describing the mechanisms of action of NS1 during viral infection have been primarily focused in IAV (Garcia-Sastre et al., 1998; Nemeroff et al., 1998; Falcon et al., 1999; Burgui et al., 2003; Twu et al., 2006; Guo et al., 2007; Kochs et al., 2007, 2009; Mibayashi et al., 2007; Hale et al., 2008a, b, 2010; Steidle et al., 2010; Dankar et al., 2013; Ayllon et al., 2014; DeDiego et al., 2016; Nogales et al., 2016b, 2017a,d, 2018a,b; Chauche et al., 2018; Rodriguez et al., 2018), IBV (Yuan and Krug, 2001; Dauber et al., 2006; Hai et al., 2008; Nogales et al., 2015; Patzina et al., 2017) and, to a less extent, ICV (Nakada et al., 1986; Muraki et al., 2010; Pachler and Vlasak, 2011). Nothing is currently known about the ability of IDV NS1 to counteract the IFN response. Moreover, it is unclear whether the NS1 proteins of IBV, ICV, or IDV have similar properties and/or mechanisms of anti-IFN activity than those of IAV NS1. Interestingly, the NS1 protein from an IAV-like virus of bat origin has been shown to also counteract the IFN system, but does not fully complement replication of IAV in mice when replacing the NS1 from a conventional IAV (Turkington et al., 2015, 2018; Aydillo et al., 2018). In a recent study, a panel of recombinant viruses expressing different NS1 from human and avian origin were generated, showing that viruses encoding NS1 from human IAV induced higher levels of IFN in human dendritic cells than viruses expressing avian IAV NS1 proteins (Monteagudo et al., 2019). Therefore, there are several functional characteristics of influenza NS1 that remains elusive.

To evaluate the ability of IBV, ICV, and IDV NS1 proteins to functionally complement IAV NS1, we engineered recombinant influenza A/Puerto Rico/8/34 H1N1 (PR8) viruses expressing homotypic (IAV) or heterotypic (IBV, ICV, and IDV) NS1 proteins. To that end, we took advantage of our recently described NS split approach that allows the expression of NS1 independently of NEP from the same NS viral segment (Manicassamy et al., 2010; Nogales et al., 2016a, b, 2017d, 2018a). Our *in vitro* and *in vivo* characterization of the recombinant PR8 viruses suggested that despite the ability of IBV, ICV, and IDV NS1 proteins to effectively counteract IFN responses *in vitro*, viral fitness *in vitro* and virulence *in vivo* were compromised in PR8 viruses expressing heterotypic (IBV, ICV, and IDV) NS1 proteins. These results indicate that while IAV, IBV, ICV, and IDV NS1 proteins are capable of counteracting host IFN responses, other functions specific for IAV NS1 are not present in the heterotypic NS1 proteins, resulting in reduced replication and viral attenuation *in vivo*. Importantly, our findings also support the feasibility of using recombinant IAV expressing heterotypic (IBV, ICV, or IDV) NS1 proteins for the development of safe and effective live-attenuated influenza vaccines (LAIVs) for the prevention of IAV infections.

## Results

### Ability of NS1 Proteins to Inhibit Host Gene Expression and Virus-Induced ISRE Promoter Activation

**FIGURE 1:**
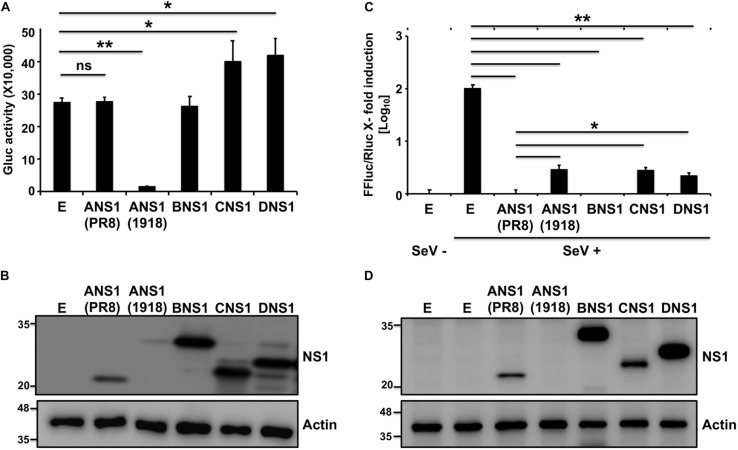
Ability of influenza NS1 proteins to inhibit host gene expression and ISRE promoter activation: Human 293T cells (2.5 × 10^5^ cells/well, 24-well plate format, triplicates) were transiently co-transfected with 25 ng of polymerase II expression pCAGGS plasmids encoding Gluc together with 1 μg of the indicated HA epitope tagged IAV (PR8 and 1918), IBV, ICV, and IDV NS1 pCAGGS plasmids, or empty (E) pCAGGS plasmid as internal control. At 24 h post-transfection, Gluc activity in the tissue culture supernatants was evaluated using a Lumicount luminometer **(A)**. Results represent the means and standard deviation (SD) of triplicate values. ^∗^*P* ≤ 0.05 using Student’s *t*-test (*n* = 3 per time point), for comparisons of cells transfected with E plasmid or plasmids encoding IAV (PR8 or 1918), IBV, ICV, or IDV NS1 proteins. **(B)** NS1 protein expression levels from transfected cells were evaluated by Western blot using an HA tag epitope antibody. Actin was used as loading control. Molecular mass markers (in kDa) are indicated on the left. **(C,D)** Ability of influenza NS1 proteins to inhibit ISRE promoter activation upon SeV infection: Human 293T cells (2.5 × 10^5^ cells/well, 24-well plate format, triplicates) were transiently co-transfected, using the CaPO_4_ method, with a reporter plasmid encoding FFluc under the control of an ISRE promoter (pISRE-FFluc), a plasmid constitutively expressing Rluc under the control of a polymerase II promoter (pSV40-Rluc), and the indicated HA epitope tagged IAV (PR8 and 1918), IBV, ICV, and IDV NS1 pCAGGS expressing plasmids, or empty (E) pCAGGS plasmid as control. At 20 h post-transfection, cells were mock infected (SeV–) or infected (SeV +) with SeV (MOI = 3) to induce activation of the ISRE promoter, and, 24 h after SeV infection, cells were analyzed for FFluc and Rluc activities using a Lumicount luminometer (**C**). Results represent the means and SD of triplicate values. ^∗^*P* ≤ 0.05 or ^∗∗^*P* ≤ 0.001 using Student’s *t*-test (*n* = 3 per time point), for comparisons of expression levels in SeV-infected cells previously transfected with E plasmid or plasmids encoding the NS1 from IAV (PR8 or 1918), IBV, ICV, or IDV. NS1 protein expression levels from total cell lysates were determined by Western blot as indicated above **(D)**. Experiments were repeated twice, with similar results.

To evaluate the ability of NS1 proteins from different influenza viruses to inhibit general host protein expression or IFN-dependent responses, we selected representative members of each viral type (IAV, IBV, ICV, and IDV). Since the NS1 protein from some IAV strains have been shown to differ in inhibiting host gene expression (Nemeroff et al., 1998; Twu et al., 2006; Hale et al., 2010; Spesock et al., 2011; Dankar et al., 2013; Ayllon et al., 2014; DeDiego et al., 2016; Nogales et al., 2016b, 2017a, 2018a; Chauche et al., 2018), we selected A/Brevig Mission/1/18 H1N1 (1918) and A/Puerto Rico/8/34 H1N1 (PR8) as representative NS1 proteins with the ability to induce or not, respectively, cellular shutoff. Moreover, the antiviral functions of these two IAV NS1 proteins have been well-described in the literature (Steidle et al., 2010; DeDiego et al., 2016; Nogales et al., 2016b, 2017a, 2018a). To assess if the NS1 proteins from different origins were able to block polymerase II promoter-driven host gene expression (Figure 1), human 293T cells were transiently co-transfected with individual plasmids encoding HA epitope tagged NS1 proteins from IAV (PR8 or 1918), IBV, ICV, or IDV; together with a plasmid expressing the Gaussia luciferase (Gluc) reporter gene (Figure 1A). Cells transfected with an empty (E) plasmid were used as an internal control in these experiments. Gluc expression was evaluated using a luminometer (Figure 1A) at 24 h post-transfection. As previously described (Steidle et al., 2010; DeDiego et al., 2016; Nogales et al., 2016b, 2017a; Chauche et al., 2018; Rodriguez et al., 2018), PR8 NS1 did not inhibit Gluc reporter gene expression, whereas 1918 NS1 was able to efficiently inhibit protein expression of the reporter gene. IBV, ICV, and IDV NS1 proteins did not inhibit Gluc expression (Figure 1A). Interestingly, a slight but statistical significant increase in the expression of Gluc was observed in cells transfected with plasmids encoding ICV and IDV NS1 proteins (Figure 1A), although the biological significance for this observation needs to be evaluated. PR8 IAV, IBV, ICV, and IDV NS1 proteins were detected by Western blot using an antibody against the HA epitope tag (Figure 1B), although different levels of expression were noted. We were not able to detect expression of 1918 NS1, mainly because of its ability to inhibit host gene expression, including its own synthesis (Figure 1B), as previously described (DeDiego et al., 2016; Nogales et al., 2016b, 2017a, 2018a). These results demonstrate that the NS1 proteins from IBV, ICV, and IDV do not inhibit host gene expression, similar to PR8 IAV NS1.

We next evaluated the ability of the different NS1 proteins to counteract IFN responses. To that end, human 293T cells were transiently co-transfected with individual plasmids expressing the HA-tagged NS1 proteins of IAV (PR8 and 1918), IBV, ICV, or IDV; together with a plasmid expressing Firefly Luciferase (FFluc) under the control of an IFN-stimulated response element (ISRE) promoter and a plasmid constitutively expressing Renilla luciferase (Rluc) under a polymerase II dependent promoter (Figure 1). Cells transfected with an empty (E) plasmid were used as a control. At 20 h post-transfection, cells were either mock infected (−) or infected (+) with Sendai virus (SeV) for 24 h to induce ISRE promoter activation. FFluc expression was quantified and normalized to the levels of Rluc (Figure 1C). While a strong activation of the ISRE promoter was observed after SeV infection in the empty plasmid control, SeV-infected cells transfected with IAV, IBV, ICV, or IDV NS1-expressing plasmids showed reduced ISRE promoter activation with values similar to those observed in mock-infected cells (Figure 1C). Moreover, PR8 IAV, IBV, ICV, and IDV; but not 1918 IAV, NS1 proteins were detected by Western blot using an anti-HA epitope antibody (Figure 1D). These results demonstrate that the NS1 proteins from IAV (PR8 and 1918), IBV, ICV, and IDV were all able to efficiently inhibit SeV-induced activation of the ISRE promoter.

### Generation and Characterization of Recombinant PR8 Viruses Expressing IAV, IBV, ICV, or IDV NS1 Proteins

**FIGURE 2:**
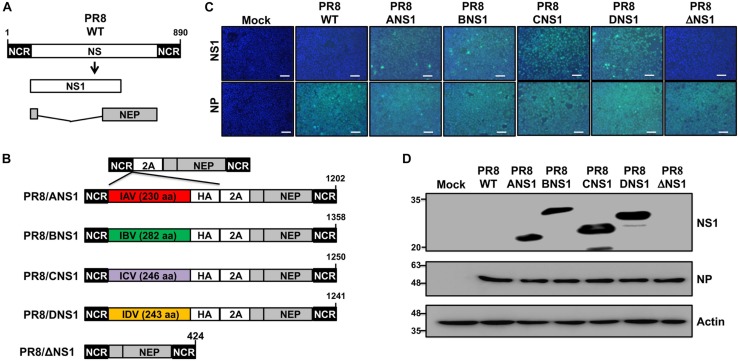
Generation and characterization of recombinant PR8 viruses expressing different NS1 proteins. **(A)** Schematic representation of PR8 WT NS segment: PR8 WT NS segment and gene products (NS1 and NEP) are indicated. **(B)** Schematic representation of PR8 viruses expressing different NS1 proteins: a modified PR8 NS segment encoding NS1 and NEP from the same transcript (top) and the PR8 viruses encoding the NS1 proteins from PR8 (PR8/ANS1), IBV (PR8/BNS1), ICV (PR8/CNS1), or IDV (PR8/DNS1) with an HA epitope tag at the C-terminal of NS1, or an PR8 NS1 deficient virus (PR8/ΔNS1) are indicated. Black boxes at the beginning and end of each viral segment represent the viral 3′ and 5′ non-coding regions (NCR). Gray boxes indicate the viral NEP. White boxes show the different NS1 proteins. The PTV-1 2A autoproteolytic cleavage site used for the expression of NS1 and NEP from a single transcript is also indicated. **(C)** Immunofluorescence assay: MDCK cells (2.5 × 10^5^ cells/well, 24-well plate format, triplicates) were mock-infected or infected (MOI = 2) with WT or modified PR8 viruses expressing IAV (PR8/ANS1), IBV (PR8/BNS1), ICV (PR8/CNS1) and IDV (PR8/DN1) NS1 proteins, or without NS1 (PR8/ΔNS1). At 14 h post-infection, cells were fixed and stained for NS1 using HA epitope tag (top) or NP (bottom) antibodies (light blue). DAPI was used for nuclear staining (dark blue). Representative images were captured at 10x magnification. Scale bar: 100 μm. **(D)** Western blots: MDCK cells (2.5 × 10^5^ cells/well, 24-well plate format) were mock-infected or infected as above and protein expression levels for NS1 and NP were evaluated using HA epitope tag or NP antibodies as described above. Actin was used as a loading control. Molecular mass markers (in kDa) are indicated on the left.

Given that all tested NS1 proteins were able to inhibit virus-mediated ISRE promoter activation in transfected cells (Figure 1), we next attempted to rescue recombinant PR8 viruses encoding homotypic IAV (PR8/ANS1) or heterotypic IBV (PR8/BNS1), ICV (PR8/CNS1), or IDV (PR8/DNS1) NS1 proteins (Figure 2). To that end, the wild-type (WT) PR8 NS segment (Figure 2A) was genetically modified to encode the viral NS1 and NEP open reading frames (ORFs) from the same viral transcript separated by the porcine teschovirus (PTV) 2A autoproteolytic cleavage site, as previously described (Manicassamy et al., 2010; Nogales et al., 2014a, 2016a,b, 2018a; Breen et al., 2016; DeDiego et al., 2016). Importantly, this construct allows us to express different NS1 sequences without affecting the amino acid sequence of NEP. We use the backbone of this new PR8 NS split segment to encode the different NS1 proteins with a C-terminal HA epitope tag (Figure 2B).

All the recombinant PR8/ANS1, PR8/BNS1, PR8/CNS1, and PR8/DNS1 viruses were successfully recovered, using plasmid-based reverse genetics techniques, and the identity of the rescued viruses was confirmed by immunofluorescence (Figure 2C) and Western blot (Figure 2D) analysis at 14 h post-infection (p.i.) in MDCK-infected [multiplicity of infection (MOI) = 2] cells, using specific antibodies against the HA epitope tag fused to NS1, and the viral NP protein. WT (PR8/WT) and NS1 deficient (PR8/ΔNS1) PR8 viruses were included as internal controls. Only cells infected with the recombinant PR8 viruses encoding the HA-tagged NS1 proteins were detected with the HA epitope tag antibody by either immunofluorescence (Figure 2C) or Western blot (Figure 2D). On the other hand, NP expression was observed in all infected but not in mock-infected cells (Figure 2D).

**FIGURE 3:**
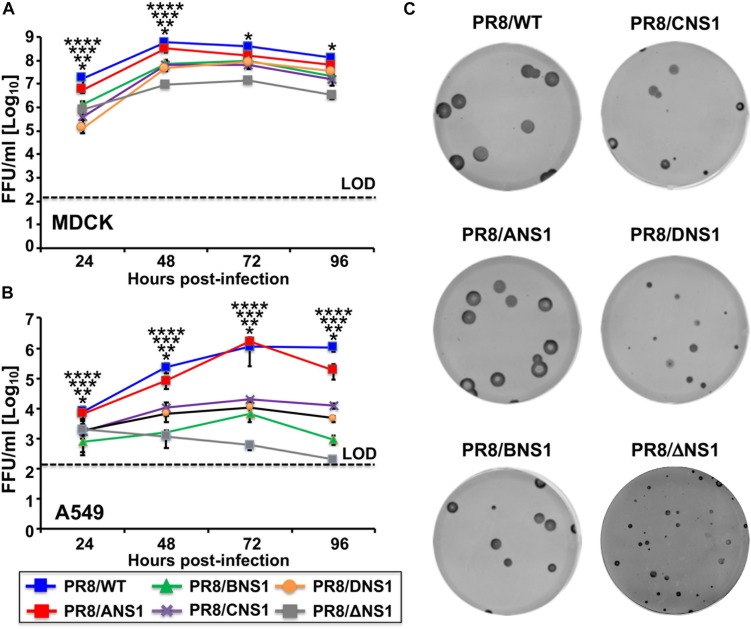
Multicycle growth kinetics and plaque assay of recombinant WT and modified PR8 viruses. Viral growth kinetics: Canine MDCK **(A)** or human A549 **(B)** cells (2.5 × 10^5^ cells/well, 24-well plate format, triplicates) were infected (MOI = 0.001 for MDCK; MOI = 0.01 for A549 cells) with the indicated WT or modified PR8 viruses and incubated at 33°C. Tissue culture supernatants were collected at the indicated hours post-infection and viral titers were determined by immunofocus assay (FFU/ml) using an anti-NP antibody (HB-65). Data represent the means and SD of the results determined from triplicate wells. Dashed lines indicate the limit of detection (200 FFU/ml). ^∗∗∗∗^, ^∗∗∗^, ^∗∗^, ^∗^*P* ≤ 0.05 (PR8/ANS1 versus PR8/BNS1, PR8/ANS1 versus PR8/CNS1, PR8/ANS1 versus PR8/DNS1, and PR8/ANS1 versus PR8/ΔNS1, respectively) using one-way ANOVA (*n* = 3 per time point). **(C)** Plaque assays: MDCK cells (10^6^ cells/well, 6-well plate format) were infected with the indicated WT or modified PR8 viruses and incubated at 33°C for 3 days. Plaques were evaluated by immunostaining with the anti-NP antibody HB-65.

Next, we evaluated the replication properties of the recombinant PR8 viruses in cell culture by performing multicycle growth kinetics in canine MDCK (MOI = 0.001) (Figure 3A) or human A549 (MOI = 0.01) (Figure 3B) cells at 33°C. All recombinant PR8 viruses reached high titers in MDCK cells, although PR8/WT and PR8/ANS1 showed statistically significant higher viral titers than PR8/BNS1, PR8/CNS1, PR8/DNS1, and PR8/ΔNS1 viruses at 24 and 48 h p.i. (Figure 3A). These similarities, or differences, were more noticeably in A549 cells (Figure 3B). PR8/WT and PR8/ANS1 reached similar viral titers in A549 cells while PR8/BNS1, PR8/CNS1, PR8/DNS1, and PR8/ΔNS1 viruses grew at significant lower titers (10- to 1,000-fold decrease) than PR8/WT or PR8/ANS1 (Figure 3B). Interestingly, while the replication of PR8/BNS1, PR8/CNS1, and PR8/DNS1 viruses increased in a time dependent matter, PR8/ΔNS1 viral titers decreased over time (Figure 3B), probably because PR8/ΔNS1 lacks NS1 and, therefore, the ability to inhibit IFN (Garcia-Sastre et al., 1998). We also examined the phenotype of the different recombinant PR8 viruses in MDCK cells using plaque assays and immunostaining (Figure 3C). As expected, based on our multicycle growth kinetics (Figure 3A), PR8/WT and PR8/ANS1 have similar plaque sizes that were larger than those of the recombinant PR8 viruses encoding heterotypic NS1 proteins, and more noticeable, than the virus lacking NS1 (PR8/ΔNS1) (Figure 3C). Altogether, these data demonstrates that viral fitness of recombinant PR8 viruses expressing heterotypic IBV, ICV and IDV NS1 proteins, and more noticeable the virus without NS1, was impaired compared to PR8/WT or PR8/ANS1 in cultured cells, an effect that was more evident in A549 cells.

### Inhibition of IFN Induction by Recombinant PR8 Viruses *in vitro*

**FIGURE 4:**
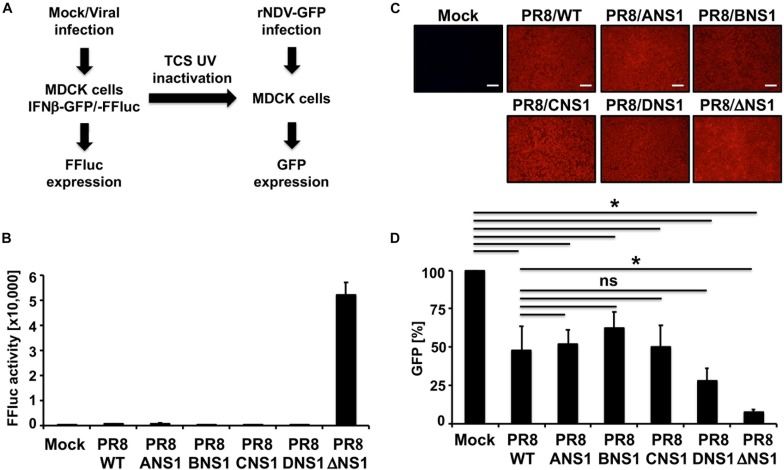
IFNβ promoter activation and induction of an antiviral state by recombinant WT and modified PR8 viruses. **(A)** Schematic representation of the experimental approach. MDCK cells (2.5 × 10^5^ cells/well, 24-well plate format, triplicates) expressing FFluc reporter gene under the control of the IFNβ promoter (MDCK IFNβ-GFP/-FFluc) were mock-infected or infected (MOI = 3) with the WT and modified PR8 viruses. At 12 h post-infection, IFNβ promoter activation was determined by assessing FFluc expression level. **(B)** Viral infections were evaluated by immunofluorescence using the antibody against influenza NP. **(C)** Tissue culture supernatants from the same mock- or PR8-infected MDCK cells were collected and, after UV inactivation, used to treat fresh MDCK cells in 96-well plates (5 × 10^4^ cells/well, 96-well plate format, triplicates) for 24 h prior to infection (MOI = 3) with rNDV-GFP. GFP expression from rNDV-GFP-infected cells was evaluated at 14 h post-infection using a fluorescent microplate reader **(D)**. ^∗^*P* ≤ 0.05 using Student’s *t*-test (*n* = 3 per time point). Ns, not significant.

Since the primary role of influenza NS1 protein is to inhibit IFN and host antiviral responses during viral infection (Hale et al., 2008b; Nogales et al., 2018b), we next explored the effect of expressing heterotypic NS1 proteins in the backbone of IAV PR8, on the ability of the virus to inhibit induction of IFN in infected cells using cell- and virus-based IFN bioassays (Figure 4A). MDCK cells constitutively expressing FFluc reporter gene under the control of the IFNβ promoter (MDCK IFNβ-GFP/IFNβ-FFluc) were mock-infected or infected (MOI = 3) with the recombinant PR8 viruses and, at 12 h p.i., we evaluated IFNβ promoter activation by FFluc expression (Figure 4B). As expected, FFluc activities were greater in cells infected with PR8/ΔNS1 (Figure 4B). However, FFluc expression levels were comparable between the PR8/WT and the homotypic (PR8/ANS1) or heterotypic (PR8/BNS1, PR8/CNS1, and PR8/DNS1) NS1-expressing recombinant viruses (Figure 4B), indicating that they were able to similarly inhibit IFNβ induction. Importantly, we observed similar levels of infection in MDCK cells with the different recombinant viruses (Figure 4C). We also assessed the presence of IFN in tissue culture supernatants (TCSs) collected from the same virus-infected MDCK cells. To that end, fresh MDCK cells were treated with the UV-inactivated TCS from PR8 infected cells. At 24 h post-incubation, cells were infected (MOI = 3) with a recombinant Newcastle disease virus expressing GFP (rNDV-GFP), a virus previously described to be sensitive to the antiviral state induced by the present of IFN in TCS (Park et al., 2003). Then, 14 h p.i., GFP expression was evaluated using a fluorescent microplate reader (Figure 4D). MDCK cells pre-treated with TCS from mock-infected cells were used as internal control. As expected, based on the levels of IFNβ promoter activation (Figure 4B), levels of rNDV-GFP replication were reduced in MDCK cells pre-treated with TCS of PR8 infected cells (Figure 4D). Importantly, inhibition of rNDV-GFP was similar in MDCK cells treated with TCS from WT (PR8/WT), homotypic (PR8/ANS1) or heterotypic (PR8/BNS1, PR8/CNS1, and PR8/DNS1) infected cells. Moreover, inhibition of rNDV-GFP infection was more evident and significant in MDCK cells treated with TCS from PR8/ΔNS1 infected cells (Figure 4D). These results demonstrate that the NS1 protein from heterotypic IBV, ICV, and IDV have similar abilities to inhibit IFN responses as the homotypic IAV PR8 NS1 in infected MDCK cells.

### Virulence of Recombinant PR8 Viruses in Mice

**FIGURE 5:**
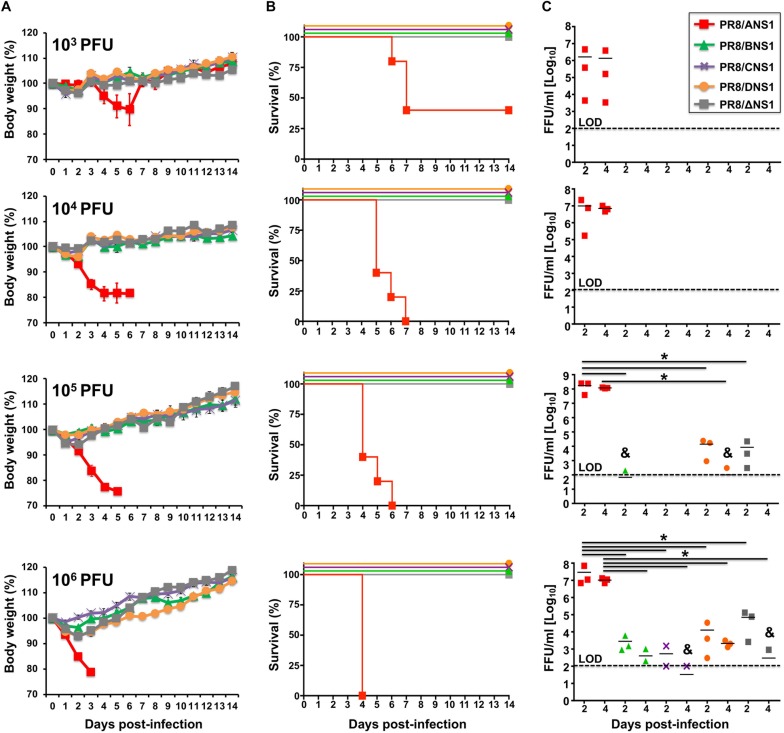
Virulence and replication of recombinant PR8 viruses in WT mice: groups of 5-to-6-week-old C57BL/6 female mice (*n* = 5) were infected intranasally with the indicated doses (10^3^, 10^4^, 10^5^, or 10^6^ PFU) of PR8 viruses expressing IAV (PR8/ANS1), IBV (PR8/BNS1), ICV (PR8/CNS1) and IDV (PR8/DNS1) or with the NS1 deficient PR8 virus (PR8/ΔNS1). Body weight loss **(A)** and survival **(B)** were evaluated daily for 2 weeks. Groups of mice (*n* = 3) were sacrificed at days 2 and 4 post-infection and lungs were harvested, homogenized and used to quantify viral titers **(C)** by immunofocus assay (FFU/ml). Symbols represent data from individual mice. Bars represent the means of lung viral titers. & Infectious virus was detected only in 1 of 3 mice. Black dotted lines indicates the limit of detection (100 FFU/ml). ^∗^*P* ≤ 0.05 using Student’s *t*-test.

Given that we observed differences in the replication of recombinant PR8 viruses expressing different NS1 proteins in A549 cells (Figure 3), we next evaluated and compared the virulence of the different recombinant PR8 viruses in a mouse model of influenza viral infection (Figure 5). Groups of C57BL/6 WT mice (*n* = 5) were infected intranasally (i.n.) with 10^3^, 10^4^, 10^5^, or 10^6^ plaque forming units (PFUs) of PR8/ANS1, PR8/BNS1, PR8/CNS1, PR8/DNS1, and PR8/ΔNS1 and animals were monitored during 2 weeks for body weight loss (Figure 5A) and mortality (Figure 5B). Mice infected with PR8/ANS1 showed clear changes in mouse morbidity and mortality that were dose dependent (Figures 5A,B, respectively). Importantly, and as previously described, approximately 50% of the mice infected with 10^3^ PFU succumbed to PR8/ANS1 infection (DeDiego et al., 2016; Nogales et al., 2016a, b). As expected, PR8/ΔNS1 was highly attenuated, with no changes in body weight and all the animals surviving viral infection, even with the highest dose of 10^6^ PFU (Figures 5A,B, respectively). Notably, PR8 viruses expressing heterotypic NS1 proteins (PR8/BNS1, PR8/CNS1, and PR8/DNS1) displayed similar levels of attenuation as PR8/ΔNS1 (Figures 5A,B), suggesting that the NS1 protein from IBV, ICV, and IDV cannot functionally substitute the NS1 protein of IAV.

To assess whether the observed attenuation correlated with virus replication in the lungs of infected mice, groups of C57BL/6 WT mice (*n* = 6) were infected i.n. with the same viral doses (10^3^, 10^4^, 10^5^, or 10^6^ PFU) of PR8/ANS1, PR8/BNS1, PR8/CNS1, PR8/DNS1, and PR8/ΔNS1 viruses; and viral titers were evaluated at days 2 (*n* = 3) and 4 (*n* = 3) p.i. (Figure 5C). Similar to the morbidity and mortality data, animals infected with PR8/ANS1 virus showed high levels of viral replication [>10^6^ fluorescent forming units (FFU/ml)] at both days 2 and 4 p.i. for all different doses (Figure 5C). Remarkably, virus replication was limited in PR8/BNS1, PR8/CNS1 and PR8/DNS1 infected mice, to levels comparable to PR8/ΔNS1 infected animals (Figure 5C). No virus was recovered in any of the mice infected at lower MOI (10^3^ and 10^4^ PFU), and low virus titers were detected in mice infected with 10^5^ PFU of PR8/BNS1 and PR8/DNS1. Different viral titers were recovered from the lungs of mice infected with the highest dose (10^6^ PFU) of PR8/BNS1, PR8/CNS1, PR8/DNS1, and PR8/ΔNS1 (Figure 5C). In all the cases, virus loads with all the four viruses (PR8/BNS1, PR8/CNS1, PR8/DNS1, and PR8/ΔNS1) were at least three to four orders of magnitude lower than those observed with PR8/ANS1 (Figure 5C). Importantly, virus titers in the lungs correlated with the low morbidity and mortality observed in infected mice (Figures 5A,B, respectively) and with viral replication in cultured cells (Figures 3A,B).

**FIGURE 6:**
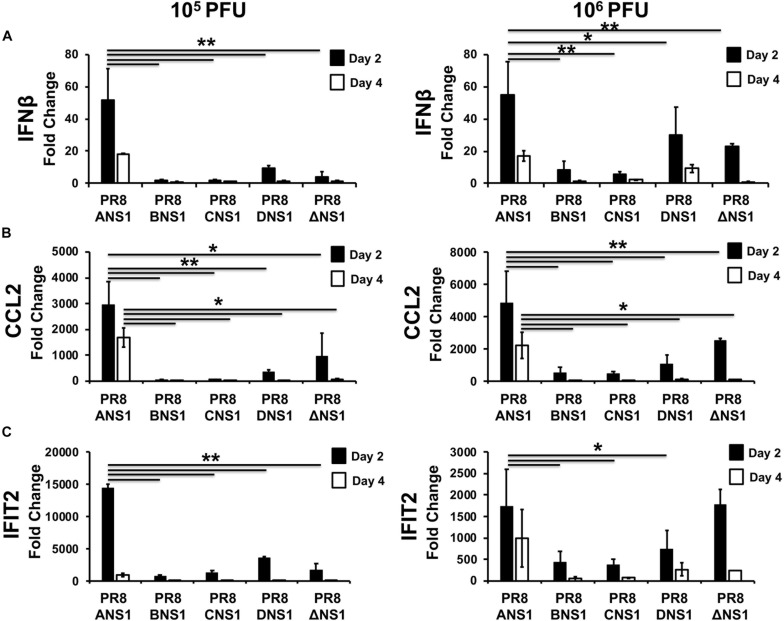
Induction of innate immune responses by recombinant PR8 viruses in WT mice: mRNA expression levels of IFNβ **(A)**, CCL2 **(B)**, and IFIT2 **(C)** in lungs of 5-to-6-week-old C57BL/6 female mice infected with 10^5^ (left) or 10^6^ (right) PFU of the indicated PR8 viruses at days 2 and 4 post-infection (*n* = 3) were quantified by RT-qPCR. Fold expression changes in each mouse group were calculated relative to the mock (PBS) control group. Data represent the averages and SD values for three mice in each group on the indicated days post-infection. ^∗^*P* ≤ 0.05 or ^∗∗^*P* ≤ 0.001 using two-way ANOVA.

In order to assess the induction of host innate immune responses after infection (Figure 6), groups of C57BL/6 WT mice (*n* = 12) were infected i.n. with 10^5^ (*n* = 6) or 10^6^ (*n* = 6) PFU of PR8/ANS1, PR8/BNS1, PR8/CNS1, PR8/DNS1, and PR8/ΔNS1; sacrificed at days 2 (*n* = 3) and 4 (*n* = 3) p.i. and IFNβ (Figure 6A), chemokine CC motif ligand 2 (CCL2) (Figure 6B), and IFN-induced protein with tetratricopeptide repeats 2 (IFIT2) (Figure 6C) mRNA expression levels were measured in the lungs by reverse transcription-quantitative PCR (RT-qPCR). PR8/ANS1 infection induced the strongest cytokine responses *in vivo* at both days p.i. with either 10^5^ or 10^6^ PFU, which most likely reflects the ability of the virus to efficiently replicate in the lungs of infected mice (Figure 6). In contrast, mice infected with PR8/BNS1, PR8/CNS1, PR8/DNS1, or PR8/ΔNS1 showed reduced levels for IFNβ (Figure 6A), CCL2 (Figure 6B), and IFIT2 (Figure 6C) mRNA expression, which also correlated with low viral replication observed in the lungs of infected mice (Figure 5).

**FIGURE 7:**
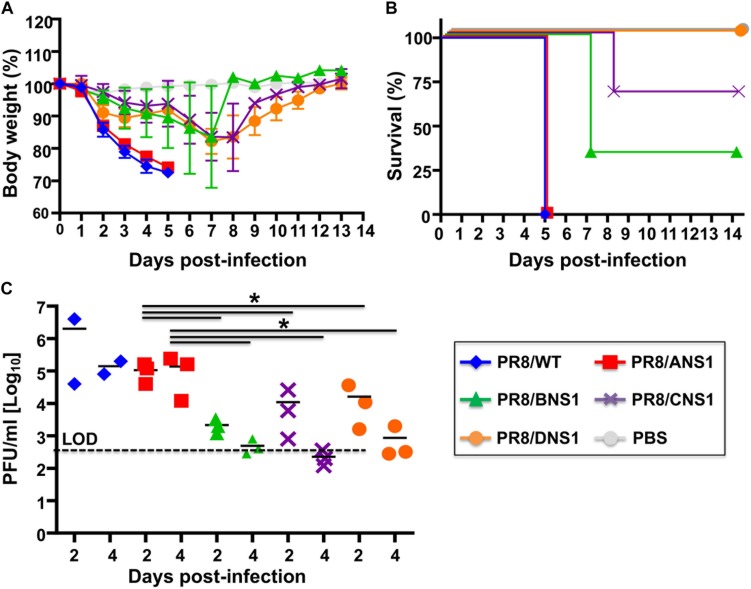
Characterization of recombinant PR8 viruses in STAT2^–/–^ mice: groups of six-to-8-week-old C57BL/6 STAT2^–/–^ mice were infected intranasally with 10^6^ PFU of the indicated recombinant PR8 viruses, or PBS as internal control. Mean body weight loss **(A)** and survival **(B)** are shown. Error bars represent SDs. **(C)** Viral titers in the lungs of animals infected with 10^6^ PFU at days 2 and 4 post-infection. Symbols represent data from individual mice. Bars represent the means of lung viral titers. The black dotted lines indicates the limit of detection (100 FFU/ml). ^∗^*P* ≤ 0.05 using Student’s *t*-test.

Signal transducer and activator of transcription 2 (STAT2) is critical for type I and type III IFN signaling and plays an important role in host defense responses against viral infections (Morrison and Garcia-Sastre, 2014; Grant et al., 2016; Uccellini and Garcia-Sastre, 2018). Phosphorylation of STAT2 (and STAT1) leads to their dimerization and translocation to the cell nucleus where they act as transcription factors for the activation of multiple ISGs, which limit viral replication and induce an antiviral state in the same or neighbor cells (Blaszczyk et al., 2016). Given that type I and type III IFN signaling relies on STAT2 activation, absence of STAT2 allows a more targeted assessment of type I and type III IFN-mediated immune responses. Moreover, it has been previously shown that influenza-infected STAT2^–/–^ B6 mice have increased morbidity and mortality when compared to WT B6 mice (Gopal et al., 2018). Therefore, we evaluated the virulence of PR8/BNS1, PR8/CNS1 and PR8/DNS1 in C57BL/6 STAT2^–/–^ mice and compared them to that of the parental PR8/WT and PR8/ANS1 viruses (Figure 7). To that end, male C57BL/6 STAT2^–/–^ mice (*n* = 3) were infected i.n. with 10^6^ PFU of PR8/WT, PR8/ANS1, PR8/BNS1, PR8/CNS1, and PR8/DNS1 or mock-infected and changes in body weight (Figure 7A) and survival (Figure 7B) were monitored daily for 14 days. All mice infected with PR8/WT or PR8/ANS1 rapidly lost weight and succumbed to viral infection by day 5 after infection. Although PR8/BNS1, PR8/CNS1, and PR8/DNS1 viruses were still attenuated as compared to PR8/WT or PR8/ANS1, the recombinant PR8 viruses expressing heterosubtypic NS1 proteins were able to induce morbidity and mortality in the STAT2^–/–^ infected mice (Figures 7A,B, respectively). To further characterize *in vivo* virulence, female STAT2^–/–^ mice were infected with 10^6^ PFU of the same recombinant viruses and viral lung replication was analyzed at days 2 and 4 p.i. (*n* = 3 for the heterotypic viruses and *n* = 2 for PR8/WT at each time point) (Figure 7C). In agreement with the pathogenicity data, lower viral titers were detected in the lungs of STAT2^–/–^ mice infected with PR8/BNS1, PR8/CNS1, or PR8/DNS1 as compared to mice infected with PR8/WT or PR8/ANS1 (Figure 7C). Altogether, these data demonstrate that although recombinant PR8 viruses expressing IBV, ICV, and IDV NS1 proteins are able to counteract IFN responses in cultured cells, these heterotypic NS1 proteins cannot entirely complement the functions of IAV NS1 *in vivo* resulting in reduced viral fitness and replication and, therefore, virulence.

## Discussion

Defense strategies provided by the innate immune system limit virus replication (Randall and Goodbourn, 2008; Carrero, 2013). However, viruses encode proteins that counteract the antiviral mechanisms of the host cell (Wang et al., 2007; Iwasaki and Pillai, 2014; Nogales et al., 2018b). Influenza NS1 protein is highly expressed in infected cells and prevents the activation of key players in the IFN system, allowing the virus to replicate and spread, representing an important viral pathogenesis factor (Garcia-Sastre et al., 1998; Hale et al., 2008b; Marc, 2014; DeDiego et al., 2016; Nogales et al., 2016b, 2018a,b). Currently, there are four types of influenza viruses: IAV, IBV, ICV and the newly identified IDV (Wright et al., 2007; Chen and Holmes, 2008; Wanitchang et al., 2012; Tong et al., 2013; Baker et al., 2014; Yoon et al., 2014; Hengrung et al., 2015; Matsuzaki et al., 2016; Wang et al., 2016; Foni et al., 2017; Nogales et al., 2017c; Su et al., 2017; Nakatsu et al., 2018; Asha and Kumar, 2019; Zhang et al., 2019). Despite the divergence of their genomes and host range, all types of influenza viruses express NS1 proteins. The NS1 protein of IAV has been extensively studied and multiple functions, including counteraction of cellular antiviral responses, have been previously described (Hatada et al., 1992, 1997; Falcon et al., 1999; Li et al., 2006; Guo et al., 2007; Kochs et al., 2007; Opitz et al., 2007; Hale et al., 2008b). Likewise, IBV NS1 has been previously shown to inhibit host innate responses, mainly IFN (Yuan and Krug, 2001; Dauber et al., 2006; Hai et al., 2008; Patzina et al., 2017). Although less studied, ICV NS1 has also been shown to inhibit IFN responses (Nakada et al., 1986; Muraki et al., 2010; Pachler and Vlasak, 2011). To date, the ability of IDV NS1 to counteract innate immune responses have not been described. Despite their similarities in inhibiting IFN, the amino acid sequence of IAV, IBV, ICV, and IDV NS1 proteins are very different, suggesting that the mechanism(s) of counteracting host innate immune responses and/or interactions with host proteins involved in the IFN pathway, could be different for the NS1 proteins of these diverse influenza viruses. The IBV, ICV, and IDV NS1 proteins tested in our study do not have the ability to inhibit host gene expression (Figure 1), similar to some IAV strains, such as PR8 (Kochs et al., 2007). However, all of them were able to inhibit ISRE promoter activation induced by SeV infection (Figure 1). This is, to our knowledge, the first time that IDV NS1 has been shown to inhibit induction of IFN responses, similar to the NS1 proteins from IAV, IBV, and ICV. Based on their ability to inhibit IFN responses, one could postulate that the NS1 protein of an influenza virus could be exchanged for that of other virus types. In this study we aimed to determine if the NS1 proteins from IBV, ICV, or IDV can functionally replace the NS1 of IAV. To address this hypothesis, we generated recombinant PR8 viruses expressing homotypic (PR8) or heterotypic (IBV, ICV, and IDV) NS1 proteins. To that end, we took advantage of our newly described IAV PR8 modified NS segment that allows the expression of NS1 independently of NEP (Breen et al., 2016; DeDiego et al., 2016; Nogales et al., 2016a, b, 2017d, 2018a) (Figure 2). Our studies suggest that although the replication of IAV expressing heterotypic IBV, ICV, and IDV NS1 proteins was not severely impaired in MDCK cells (Figure 3) and the viruses were able to inhibit IFN response to levels comparable to PR8/WT (Figure 4), viral replication was impaired in human A549 cells (Figure 3). In addition, our results *in vivo* showed that virulence and replication of IAV expressing heterotypic NS1 proteins were also affected in WT mice (Figure 5). Notably, the virulence and replication of PR8 viruses expressing heterotypic NS1 proteins were still maintained, although to a minor extent, in STAT2^–/–^ mice (Figure 7), suggesting that although IBV, ICV, and IDV NS1 proteins are able to counteract IFN responses similar to IAV NS1 *in vitro*, they cannot complement additional functions that are required for viral replication and virulence.

IAV NS1, through its N-terminal domain, is able to bind multiple RNA molecules (Marc, 2014), including polyadenylated mRNAs, double stranded (ds)RNAs, small nuclear RNAs, viral genomic RNAs or messenger (m)RNAs (Hatada et al., 1992, 1997; Lu et al., 1994; Qiu and Krug, 1994; Qiu et al., 1995; Marion et al., 1997; Wang and Krug, 1998; Terrier et al., 2013). In addition, more than 50 host proteins have been described to interact with IAV NS1 (Marc, 2014; Nogales et al., 2018b), although there is some variability among the NS1 binding partners between different IAV strains, most likely due to host-adaptation mechanisms (Nogales et al., 2017a, 2018a,b; Chauche et al., 2018; Rodriguez et al., 2018). Furthermore, IAV NS1 is also able to interact with viral proteins such as the polymerase acid (PA) or NP (Robb et al., 2011; Marc, 2014). Through these numerous interactions, IAV NS1 is able to inhibit antiviral responses and translation, regulate the activity of the viral polymerase complex and, therefore, synthesis of vRNA; and control several processes related with the cellular or vRNA metabolism (RNA splicing, mRNA nuclear export, etc.). Currently, knowledge on the ability of IBV, ICV, and IDV NS1 proteins to bind RNA or cellular proteins is more limited, and these differences could explain the attenuation of PR8 viruses expressing heterotypic NS1 proteins.

Moreover, our results have also an important translational application. Since recombinant IAV expressing heterotypic (IBV, ICV, or IDV) NS1 proteins are highly attenuated, with limited replication *in vivo* as compared to viruses expressing the homotypic IAV NS1 protein, these viruses could be used as safe, immunogenic and protective LAIVs for the prevention of viral infections. Compared to the previously described approaches, including the use of a NS1 deficient virus, LAIVs based on IAV expressing heterotypic NS1 proteins present the following advantages: (1) IAV expressing heterotypic NS1 proteins replicate more efficiently in culture cells than NS1 deficient viruses, representing an advantage for vaccine production; (2) IAV expressing heterotypic NS1 proteins would ensure that the viruses are attenuated *in vivo* but they conserve their ability to counteract, to some extent, the IFN response; and (3) therefore, they could induce innate immune responses that can act as a natural adjuvant to control viral replication and to enhance host adaptive immune response to provide better protection (Fernandez-Sesma et al., 2006; Fernandez-Sesma, 2007; Mueller et al., 2010).

Altogether, our data suggest that despite the similarities to effectively counteract IFN responses, the NS1 proteins of IBV, ICV, or IDV do not fully complement the activities of the multifunctional IAV NS1 protein. Our results also open the feasibility of conducting similar studies in the backbone of other influenza types (e.g., IBV, ICV, and IDV) to evaluate the contribution of their NS1 proteins in viral pathogenesis and in the feasibility of complementing them with the NS1 protein from heterotypic viruses.

## Materials and Methods

### Cell Lines and Viruses

Human embryonic kidney 293T (American Type Culture Collection, ATCC, CRL-11268), human lung epithelial carcinoma A549 (ATCC CCL-185) and Madin-Darby canine kidney (MDCK, ATCC CCL-34) cells were grown in Dulbecco’s modified Eagle’s medium (DMEM; Mediatech, Inc.) supplemented with 5% fetal bovine serum (FBS; Atlanta Biologicals) and 1% penicillin (100 units/ml)–streptomycin (100 μg/ml)–2 mM L-glutamine (P-S-G; Mediatech, Inc.) at 37°C in air enriched with 5% CO_2_. MDCK cells constitutively expressing the green fluorescent protein (GFP) and firefly luciferase (FFluc) reporter genes under the control of the IFNβ promoter (MDCK IFNβ-GFP/IFNβ-FFluc) were previously described (Hai et al., 2008). Influenza A/Puerto Rico/8/1934 (PR8) H1N1 wild-type (WT) and ΔNS1 viruses, Cantell strain of Sendai virus (SeV), and the recombinant Newcastle disease virus (rNDV) expressing GFP (rNDV-GFP) were also previously described (Garcia-Sastre et al., 1998; Park et al., 2003; Martinez-Sobrido et al., 2006; Kochs et al., 2007; Cox et al., 2015; Nogales et al., 2016a).

### Plasmids

Polymerase II expression plasmids containing the NS1 sequences from influenza B/Brisbane/60/2008 (IBV), C/Taylor/1233/1947 (ICV), or D/Swine/Oklahoma/1334/2011 (IDV) fused to an HA epitope tag (YPYDVPDYA) were generated using standard molecular biology techniques. Briefly, the different NS1 ORFs were amplified by RT-PCR using oligonucleotides with the appropriate flanking restriction sites (*Sac*I and *Sma*I) for cloning into pCAGGS-HA-COOH (Mibayashi et al., 2007) using *Sac*I and *Sma*I restriction sites. The pCAGGS plasmids encoding the NS1 genes of IAV PR8 and A/Brevig Mission/01/1918 H1N1 (1918) have been previously described (Kochs et al., 2007; Mibayashi et al., 2007).

In order to generate ambisense pDZ-NS plasmids containing the different IAV (PR8), IBV, ICV, and IDV NS1 proteins and the same IAV PR8 NEP, we used our previously described pDZ-NS-2xBsmBI plasmid (Nogales et al., 2014a), which contains the PR8 NS1, without the stop codon or splice acceptor site, and two *Bsm*BI sites, followed by the porcine teschovirus-1 (PTV-1) 2A autoproteolytic cleavage site (ATNFSLLKQAGDVEENPGP) and the PR8 NEP. The PR8 NS1 was removed from the pDZ-NS-2xBsmBI plasmid by inverse PCR to generate pDZ-ΔNS1-2xBsmBI. Next, the different IBV, ICV, or IDV ORFs were amplified by PCR from the pCAGGs-NS1 plasmids with primers containing *Bsm*BI sites and an HA tag at the C-terminal. NS1 PCR products were cloned into the pDZ-ΔNS1-2xBsmBI plasmid using the *Bsm*BI restriction sites. All the plasmid constructs were confirmed by sequencing (ACGT, Inc.). Sequences of the primers used for the construction of the different plasmids are available upon request.

### Rescue of Recombinant PR8 Viruses

Ambisense pDZ plasmids were used for the rescue of the recombinant PR8 viruses as previously described (Nogales et al., 2014b, 2016a; Breen et al., 2016; Nogales and Martinez-Sobrido, 2016). Briefly, co-cultures (1:1) of 293T/MDCK cells (6-well plate format, 10^6^ cells/well) were co-transfected in suspension, using Lipofectamine 2000 (LPF2000, Invitrogen), with the PR8 ambisense pDZ-PB2, -PB1, -PA, -HA, -NP, -NA, -M plasmids and the NS plasmids containing IAV (PR8), IBV (B/Brisbane/60/2008), ICV (C/Taylor/1233/1947) or IDV (D/Swine/Oklahoma/1334/2011) NS1 proteins. Clonal viruses were selected by plaque assay, and virus stocks were propagated in MDCK cells at 33°C in a 5% CO_2_ atmosphere for 3–4 days. For infections, virus stocks were diluted in phosphate buffered saline (PBS) supplemented with 0.3% bovine albumin (BA) and 1% PS (PBS/BA/PS). After viral infections, cells were maintained in DMEM supplemented with 0.3% BA, 1% PSG, and 1 μg/ml tosyl-sulfonyl phenylalanyl chloromethyl ketone (TPCK)-treated trypsin (Sigma). Virus titers were determined by standard plaque assay (plaque forming units [PFU]/ml) in MDCK cells (Nogales et al., 2014b, 2016a; Breen et al., 2016; Nogales and Martinez-Sobrido, 2016).

### Inhibition of Host Gene Expression

To evaluate the effects of the different viral NS1 proteins on host protein synthesis, human 293T cells (24-well plate format, 2.5 × 10^5^ cells/well, triplicates) were transiently co-transfected, using LPF2000, with 1 μg/well of the indicated pCAGGS-HA COOH NS1 expression plasmids or an empty plasmid as control, together with 25 ng/well of pCAGGS plasmid expressing Gaussia luciferase (Gluc) (Capul and de la Torre, 2008). At 24 h post-transfection, Gluc activity was quantified from tissue culture supernatants using a Biolux Gaussia luciferase reagent (New England Bio-Labs) and a Lumicount luminometer (PacKard). The mean values and standard deviations (SD) were calculated using Microsoft Excel software.

### Inhibition of ISRE Promoter Activation

To evaluate the ability of the different NS1 proteins to inhibit activation of an IFN-stimulated response element (ISRE) promoter upon SeV infection, human 293T cells (24-well plate format, 2.5 × 10^5^ cells/well, triplicates) were co-transfected with 1 μg of the different pCAGGS-HA NS1-expressing plasmids or empty plasmid as a control, together with 0.25 μg/well of a plasmid expressing FFluc under the control of an ISRE promoter (pISRE-FFluc) (Kochs et al., 2007; Nogales et al., 2017a; Rodriguez et al., 2018), and a plasmid constitutively expressing Renilla luciferase under a constitutively expressing simian virus 40 promoter (pSV40-Rluc) using a calcium phosphate-based mammalian transfection kit (Stratagene). At 20 h post-transfection, cells were infected (MOI = 3) with SeV, Cantell strain (Kochs et al., 2007; Nogales et al., 2017a; Rodriguez et al., 2018), to induce activation of the ISRE promoter, and 24 h post-infection, FFluc and Rluc activities were quantified from cell lysates using luciferase reporter buffers (Promega) and a Lumicount luminometer (PacKard). FFluc/Rluc activation was shown as fold-induction compared to mock-infected cells in the absence of NS1 protein (empty plasmid). The mean values and SD were calculated using Microsoft Excel software.

### SDS-PAGE and Western Blot Analysis

Total proteins from transfected or infected cell lysates were separated by denaturing electrophoresis in 10% SDS-polyacrylamide gels and transferred to nitrocellulose membranes (Bio-Rad). Membranes were blocked for 1 h with 5% dried skim milk in PBS containing 0.1% Tween 20 (PBS-Tween) and incubated overnight at 4°C with rabbit polyclonal anti-HA (Sigma; to detect expression of NS1) or mouse monoclonal anti-NP (HB-65; ATCC H16-L10-4R5) antibodies. An anti-β-actin monoclonal antibody (Sigma) was used as a loading control. Horseradish peroxidase (HRP) secondary antibodies (GE Healthcare) specific for mouse or rabbit immunoglobulins (Ig) were used to detect bound primary antibodies. Proteins were detected by chemiluminescence using the Super Signal West Femto maximum-sensitivity substrate (Thermo Scientific) following the manufacturer’s instructions.

### Immunofluorescence Assays

MDCK cells were mock infected or infected (MOI = 3) with the indicated PR8 viruses. At 14 h post-infection, cells were fixed with 4% paraformaldehyde (PFA) and permeabilized with 0.5% Triton X-100 in PBS for 15 min at room temperature. Immunostaining was performed as described previously (Nogales et al., 2014b, 2016a; Breen et al., 2016; Nogales and Martinez-Sobrido, 2016), using primary rabbit polyclonal anti-HA (Sigma; to detect NS1 expression) or mouse monoclonal anti-NP (HB-65) antibodies and FITC-conjugated anti-mouse or anti-rabbit secondary antibodies (Dako). Cell nuclei were stained with 4′,6′-diamidino-2-phenylindole (DAPI, Research Organics). Images were taken with a fluorescence microscope (Nikon Eclipse TE2000).

### Virus Growth Kinetics

To assess virus multicycle growth rates, confluent monolayers MDCK or A549 cells (12-well plate format, 5 × 10^5^ cells/well, triplicates) were infected at a MOI of 0.001 or 0.01, respectively. After 1 h of virus adsorption at room temperature, cells were incubated in DMEM supplemented with 0.3% BSA, PSG, and TPCK-treated trypsin at 33°C. At the indicated times post-infection (24, 48, 72, and 96 h), tissue culture supernatants were collected and titrated on MDCK cells in 96-well plates by an immunofluorescence assay (fluorescent forming units [FFU/ml]) using an influenza virus anti-NP antibody (HB-65) as previously described (Nogales et al., 2014a, b). The mean values and SD were calculated using Microsoft Excel software.

### Plaque Assays

Confluent monolayers of MDCK cells (6-well plate format, 10^6^ cells/well) were infected with the indicated PR8 viruses for 1 h at room temperature, and after virus adsorption, cells were overlaid with media containing agar and incubated at 33°C. At 3 days post-infection, cells were fixed with 4% paraformaldehyde for 1 h at room temperature. Then, the overlays were removed, cells were permeabilized (0.5% Triton X-100 in PBS) for 15 min at room temperature, and immunostained, as previously described (Nogales et al., 2017a, d), using the anti-NP antibody HB-65 and vector kits (Vectastain ABC kit and DAB HRP substrate kit; Vector) according to the manufacturer’s specifications.

### *In vivo* Experiments

Studies with C57BL/6 WT or STAT2^–/–^ were conducted at the University of Rochester or Icahn School of Medicine at Mount Sinai, respectively.

All protocols involving C57BL/6 WT mice have been approved by the University of Rochester Committee of Animal Resources and complied with the recommendations in the Guide for the Care and Use of Laboratory Animals of the National Research Council (National Research Council (U.S.) Committee for the Update of the Guide for the Care and Use of Laboratory Animals et al., 2011). C57BL/6 WT were purchased from the National Cancer Institute (NCI) and maintained in the animal care facility at University of Rochester under specific pathogen-free conditions. Six-to-8-week-old C57BL/6 WT mice were anesthetized intraperitoneally (i.p.) with 2,2,2-tribromoethanol (Avertin; 240 mg/kg of body weight) and infected intranasally (i.n.) with the indicated viruses. Mice (*n* = 5) were examined daily for morbidity (body weight loss) and mortality (survival). Mice showing a > 25% loss of their initial body weight were considered to have reached the experimental endpoint and were humanely euthanized. Virus replication was assessed by evaluating viral titers in the lungs of infected mice at days 2 and 4 post-infection. To that end, C57BL/6 WT mice (*n* = 3/day) were euthanized by administration of a lethal dose of Avertin and bleeding, and lungs were surgically extracted and homogenized in 1 ml of PBS/BA/PS (Rodriguez et al., 2017). Virus titers were determined by immunofocus assay (fluorescent forming units [FFU/ml]) in MDCK cells as previously described (Nogales et al., 2014a, b, 2015, 2016a; Cox et al., 2015; Breen et al., 2016).

Levels of IFNβ, chemokine (C–C) motif ligand 2 (CCL2), and IFN-induced protein with tetratricopeptide repeats 2 (IFIT2) induction were analyzed in mouse lungs at 2 and 4 days post-infection. To that end, mice (*n* = 3) were sacrificed as above, and their lungs were extracted and incubated in RNAlater (Ambion) at 4°C for 24 h prior to freezing at −80°C. Lungs were homogenized in lysis buffer by use of a gentleMACS dissociator (Miltenyi Biotec), and total RNA was extracted with an RNeasy minikit (Qiagen) according to the manufacturer’s recommendations. Reverse transcriptase reactions were conducted at 37°C for 2 h using a High-Capacity cDNA reverse transcription kit (Applied Biosystems) and a dT primer to amplify mRNAs. Quantitative PCRs (qPCRs) were performed using TaqMan gene expression assays (Applied Biosystems) specific for the murine genes IFNβ (Mm00439552_s1), IFIT2 (Mm00492606_m1), and CCL2 (Mm00441242_m1). The 2^–Δ^ ^Δ^ ^CT^ method was used for quantification, and values were presented as fold induction in infected mice compared to non-infected mice (Livak and Schmittgen, 2001).

STAT2^–/–^ C57BL/6 mice were originally provided by Dr. Christian Schindler. The latter STAT2^–/–^ mice were then bred locally. Mice were housed in a barrier facility at the Icahn School of Medicine at Mount Sinai under specific pathogen free conditions. All mouse procedures were approved by the Institutional Animal Care and Use Committee (IACUC) of the Icahn School of Medicine at Mount Sinai and performed in accordance with the IACUC guidelines. Six-to-8-week-old C57BL/6 STAT2^–/–^ mice were anesthetized by inhalation of 4% isoflurane and inoculated i.n. at the indicated amount of viruses. Mice (*n* = 5 for WT or *n* = 3 for STAT2^–/–^) were examined daily for morbidity (body weight loss) and mortality as indicated above. To evaluate viral replication, C57BL/6 STAT2^–/–^ mice (*n* = 3 for ABCD NS1-expressing recombinant viruses, *n* = 2 for PR8/WT virus) were euthanized by inhalation of 2% CO_2_ followed by cervical dislocation. The lungs were excised on day 2 or day 4 post-infection, and viral titers were determined by plaque assay in MDCK cells. Mice were distributed by sex because of limited availability. Male mice were used to evaluate morbidity and female mice were used to assess viral replication.

## Data Availability Statement

All datasets generated for this study are included in the article/supplementary material.

## Ethics Statement

The animal study was reviewed and approved by Studies with C57BL/6 WT or STAT2–/– were conducted at the University of Rochester or Icahn School of Medicine at Mount Sinai, respectively. All protocols involving C57BL/6 WT mice have been approved by the University of Rochester Committee of Animal Resources and complied with the recommendations in the Guide for the Care and Use of Laboratory Animals of the National Research Council (National Research Council (U.S.) Committee for the Update of the Guide for the Care and Use of Laboratory Animals et al., 2011). All mouse procedures were approved by Institutional Animal Care and Use Committee (IACUC) of the Icahn School of Medicine at Mount Sinai and performed in accordance with the IACUC guidelines.

## Author Contributions

LM-S, AN, and AG-S conceived the experiments. AN, TA, GÁ-P, AE, KC, RC, and MD conducted the experiments. AN and LM-S wrote the manuscript. FL provided critical reagents. All authors critically reviewed the manuscript.

## Conflict of Interest

AG-S is a named co-inventor on technology patents own by Icahn School of Medicine at Mount Sinai (ISMMS) related to using live attenuated influenza virus vaccines lacking the NS1 virulence gene, exclusively licensed to Vivaldi Biosciences, Inc. AG-S is a co-founder of and holds equity in Vivaldi Biosciences, Inc., a privately-held start-up company in which ISMMS also holds equity. The remaining authors declare that the research was conducted in the absence of any commercial or financial relationships that could be construed as a potential conflict of interest.
